# Over-expressing Akt in T cells to resist tumor immunosuppression and increase anti-tumor activity

**DOI:** 10.1186/s12885-015-1611-4

**Published:** 2015-08-27

**Authors:** Yanhong Wu, Zhenling Deng, Yishu Tang, Shuren Zhang, Yu-Qian Zhang

**Affiliations:** Department of Immunology, Cancer Hospital & Institute, Peking Union Medical College and Chinese Academy of Medical Sciences, Beijing, 100021 China

## Abstract

**Background:**

Tumor employs various means to escape immunosurveillance and inhibit immune attack, and strategies have been developed to counteract the inhibitory signals. However, due to the complex suppressive mechanisms in the tumor microenvironment, blocking one or a few inhibitory signals has only limited effects on therapeutic efficacy. Instead of targeting tumor immunosuppression, we considered from another point of view, and hypothesized that manipulating T cells to make them resist any known or unknown suppressive mechanism may be more effective for cancer treatment.

**Methods:**

We used OT-1 cells transduced with retroviruses encoding Akt and human peripheral blood lymphocytes (PBLs) transduced with retroviruses encoding both Akt and a chimeric antigen receptor (CAR) specific for tumor antigen EpCAM to examine the effect of over-expressing Akt on tumor specific T cells in tumor environment.

**Results:**

We show that Akt activity of T cells in the tumor environment was inhibited, and over-expressing Akt in OT-1 cells increased the cytokine production and cell proliferation in the presence of B16-OVA tumor cells. What’s more, adoptive transfer of OT-1 cells over-expressing Akt inhibited B16-OVA tumor growth and prolonged mouse survival. To examine if over-expressing Akt could increase the anti-tumor activity of T cells in human cancer, PBLs co-expressing EpCAM specific CAR and Akt were cultured with EpCAM-expressing human prostate cancer cells PC3M, and less inhibition on cell proliferation and less apoptosis were observed. In addition, adoptive transfer of PC3M specific T cells over-expressing Akt resulted in more dramatic tumor inhibitory effects in PC3M bearing NOD/SCID mice.

**Conclusions:**

These data indicates that over-expressing Akt in tumor specific T cells increases T cell proliferation and activity in the tumor environment, and enhances anti-tumor effects of adoptively transferred T cells. Our study provides a new strategy to improve the efficacy of adoptive T cell therapy, and serves as an important foundation for clinical translation.

**Electronic supplementary material:**

The online version of this article (doi:10.1186/s12885-015-1611-4) contains supplementary material, which is available to authorized users.

## Background

Tumor immunosuppressive microenvironment is the major obstacle for successful clinical translation of immunotherapeutic approaches. Tumor employs different strategies to escape immunosurveillance, including impairment of the antigen presentation, up-regulating negative co-stimulatory signals, secretion of immunosuppressive factors, activation of pro-apoptotic pathways, and recruitment of different regulatory cell populations [1, 2]. By these various means, tumor induces a complex immunosuppressive microenvironment to evade immune response and restrict the effectiveness of cancer vaccine and adoptive transfer of tumor specific T cells.

With deeper understanding of the interactions between tumor and immune system, therapeutic strategies have been developed to resist immunosuppression, such as using antibodies to block CTLA-4 or PD-1 signaling, inhibiting IDO activity, depleting regulatory T cells, etc. [3]. However, it’s easy to understand that, confronting such a complex immunosuppressive microenvironment, strategies targeting one or two inhibitory signals have only limited effects on therapeutic efficacy. Instead of dealing with multiple inhibitory factors, we considered if there is any means to manipulate effector T cells to make them resist any known or unknown immunosuppressive mechanism. Through analysis of T cell signaling pathways, we found that Akt is in the central node of immune modulation.

The serine/threonine kinase Akt (PKB) is utilized in a variety of signaling pathways from T cell growth factors such as IL-7R, and CD28 co-stimulatory signal [4, 5]. CD28 activation enables recruitment and activation of phosphatidylinositol 3-kinase (PI3K), resulting in the generation of phosphatidylinositol-3,4,5-trisphosphate (PIP3), which recruits pleckstrin homology (PH) domain containing proteins including Akt to the plasma membrane. After recruitment to the plasma membrane, Akt becomes phosphorylated and activated by PDK1, and then plays an important role in diverse cellular processes including cell survival, glucose metabolism, and cytokine synthesis [6–8]. Besides co-stimulatory receptors, co-inhibitory receptors also regulate Akt activation. Ligation of CTLA-4 and PD-1 both inhibit Akt activity, suggesting PI3K-Akt signaling is a major mechanism of immune regulation [9, 10]. Consistent with this, it has been reported that T cells expressing constitutively active Akt displayed increased viability in the absence of stimulation, and could grow rapidly and secrete cytokines in the absence of CD28 co-stimulation [11].

Based on these findings, we hypothesize that up-regulating Akt activity in tumor specific T cells could help T cells resist tumor immunosuppression and improve the anti-tumor effects of adoptive immunotherapy. To test this hypothesis, we used two different tumor models, B16-OVA tumor model and human prostate cancer PC3M tumor model, and demonstrated that over-expressing Akt in tumor specific T cells could increase T cell proliferation and cytokine secretion when co-cultured with tumor cells, and inhibit tumor growth *in vivo*. Our data suggests over-expressing Akt in effector T cells as a new strategy to improve the efficacy of adoptive T cell transfer for cancer.

## Methods

### Mice

OT-1 transgenic mice were purchased from the Jackson Laboratory, C57BL/6 mice and NOD/SCID mice were purchased from Vital River Laboratories. All mice were maintained under specific pathogen-free conditions in the animal facility of Cancer Institute, Chinese Academy of Medical Sciences (CAMS), and all procedures for animal experiments were approved by the Institutional Animal Use and Care Committee of CAMS.

### Cell lines

The OVA-expressing tumor cell line, B16-OVA, was kindly provided by T.-C. Wu lab (Johns Hopkins Medical Institutions, Baltimore, MD) [12], and cultured in RPMI1640 supplemented with 10 % FBS, 2 mM L-glutamine, 1 mM sodium pyruvate, 2 mM non-essential amino acids, 50 units/ml penicillin and streptomycin. PC3M cell line was purchased from ATCC, and maintained in culture with DMEM medium (Gibco) supplemented with 10 % FBS.

### Retroviral vector construction and retrovirus preparation

PLNCX-myr-HA-Akt1 (William Sellers, Addgene plasmid 9005) and pLNCX-HA-Akt1 (William Sellers, Addgene plasmid 9004) were obtained from Addgene [13]. PLNCX empty vector was used as the control retrovirus. To construct EpCAM specific CAR and Akt co-expressing construct, HindIII/HpaI/BglII-IRES-XhoI/SalI/ClaI sequence was synthesized (IRES sequence corresponds to 1119–1693 of pIRES-G, with 1346G → C) and inserted into HindIII/ClaI multiple cloning sites of pLNCX retroviral vector to make pLNCX-IRES. EpCAM specific CAR consisting of an anti-EpCAM scFv (sequence corresponds to Genebank identifier AJ564232.1) [14], part of the extracellular domain and the entire transmembrane and intracellular domains of CD28, and the cytoplasmic domain of CD3ζ (the CD28 and CD3ζ sequence included corresponds to Genebank identifier HM852952.1) [15] was synthesized and inserted into HindIII/BglII of pLNCX-IRES to make pLNCX-CAR. Akt and myr-Akt were cloned from pLNCX-HA-Akt1 and pLNCX-myr-HA-Akt1 respectively, and inserted into XhoI/ClaI of pLNCX-CAR to make pLNCX-CAR-Akt and pLNCX-CAR-myr-Akt co-expressing constructs. Retroviruses were prepared using Retrovirus Packaging Kit Ampho (TaKaRa) and 293 T packaging cell line, following the manufacturer’s instruction.

### Retroviral transduction of OT-1 cells and human PBLs

Splenocytes and lymph node cells of OT-1 transgenic mice were harvested, and red blood cells were removed by RBC lysis buffer (Biolegend). CD8^+^ T cells were isolated using mouse CD8a^+^ T cell isolation kit (Miltenyi Biotec), and then were stimulated with 1 ug/ml anti-mouse CD3 and 1 ug/ml anti-mouse CD28 antibodies (Biolegend) for 2 days at 1 × 10^6^ cells/ml in RPMI medium (Gibco) supplemented with 10 % FBS, 1x nonessential amino acid, L-glutamine, sodium pyruvate, and penicillin-streptomycin, and 0.1 % β-mercaptoethanol. For retrovirus transduction, 24-well plates were coated with RetroNectin (TaKaRa) at 4 °C overnight according to the product’s manual, and blocked with 2 % BSA at room temperature for 30 min. After that, the plates were added with retrovirus supernatants at 300 ul/well and incubated at 37 °C for 6 h. Then the stimulated cells were transferred to the pre-coated wells at 1 × 10^6^ cells/ml and cultured at 37 °C for 5 days in the presence of 100 U/ml recombinant human IL-2.

For PBL preparation, donor blood was obtained from healthy volunteers with consent from institutional review board of Cancer Institute, Chinese Academy of Medical Sciences, and written informed consent for participation in the study was obtained from participants. PBMCs were prepared by centrifugation of the blood on Ficoll-Hypaque density gradients (Sigma-Aldrich), and adherent cells were removed by plating in cell culture plates for 2 h. Then the cells were stimulated with 1 ug/ml OKT3 and 1 ug/ml anti-human CD28 antibodies (Biolegend) at 1 × 10^6^ cells/ml for 2 days, and transduced with retroviruses as described above.

### Western blot

Cell lysis was performed on ice for 30 min in RIPA buffer with 1x protease inhibitor cocktail, 0.5 mM PMSF, 30 mM NaF, 1 mM Na_3_VO_4_ added freshly. After centrifugation at 1000 rpm at 4 °C for 10 min, the protein concentration of the supernatant was determined by BCA assay, and 40 ug of protein extract was subjected to SDS-PAGE and western blot following standard procedures. Phospho-Akt (Ser473) Rabbit mAb (Cell Signaling Technology), Akt (pan) Rabbit mAb (Cell Signaling Technology), and β-actin antibody (Santa Cruz Biotechnology) were used at 1:2000 to probe the western blots.

### RT-PCR and quantitative real-time PCR

Total RNA was extracted using the RNeasy Mini kit (Qiagen) and was reverse transcribed using ReverTra Ace qPCR RT Master Mix with gDNA Remover (TOYOBO). PCR was performed with EmeraldAmp MAX PCR Master Mix (TAKARA), and the following primers were used: CAR, 5′- CCTTATGGTTACGACGAGTATGGTCTGG and 3′- AACAGTTTAGGAGGCTGCCCTGGTTTCT; β-actin, 5′-AAGAGAGGCATCCTCACCCT and 3′- TACATGGCTGGGGTGTTGAA. Real-time PCR was performed with iTaq Universal SYBR Green Supermix (BIO-RAD) using CFX96 Touch Real-Time PCR Detection System (BIO-RAD), and the primers were purchased from GeneCopoeia (Catalog# HQPCS0002).

### Flow cytometry

Cells were stained with FITC or PE-conjugated CD8, IFN-γ, HLA-DR, CD80, B7-H1, TGF-β, IL-10 antibodies following standard protocol. All antibodies were purchased from Biolegend. CFSE cell proliferation kit (Life Technologies) was used to determine cell proliferation, and APC Annexin V apoptosis detection kit with PI (Biolegend) was used to detect cell apoptosis. Cells were analyzed by a FACS Calibur flow cytometer and Flowjo software.

### Luminescence imaging

Luciferase expressing cell line PC3M-luc was established by transducing PC3M with retroviruses encoding luciferase and selection with G418. To examine the cytotoxicity of PBLs transduced with different retroviruses, PC3M-luc cells were incubated with effector cells for 24 h, and then luciferin (Promega) was added at a final concentration of 0.3 mg/ml. 10 min later, luminescence imaging was conducted on IVIS system (Xenogen/Caliper Life Sciences).

### Statistical analysis

Data are presented as mean ± SE. To determine the significance of differences between samples or groups, the student’s *t*-test or two-way ANOVA was used as indicated in the figure legends.

## Results

### Akt expression in the T cells is down-regulated in the tumor microenvironment

**Fig. 1:**
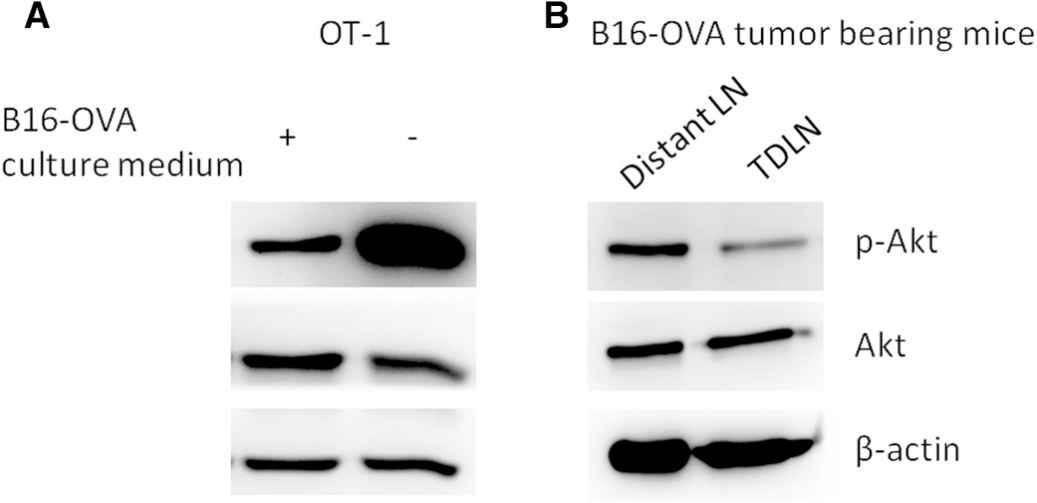
Akt activity of T cells is down-regulated in tumor environment. **a** CD8^+^ T cells isolated from OT-1 transgenic mice were stimulated with anti-CD3 and anti-CD28 antibodies for 2 days, and then incubated with or without B16-OVA tumor cell culture supernatant. 24 h later, cells were collected, and the levels of Akt, phosphorylated Akt, and β-actin were examined by western blot. **b** CD8^+^ T cells were isolated from tumor draining lymph nodes (TDLN) or distant lymph nodes of B16-OVA tumor bearing mice, the levels of Akt, phosphorylated Akt, and β-actin were examined by western blot

To verify that Akt activation in T cells is inhibited in tumor environment, T cells were isolated from OT-1 transgenic mice, stimulated with anti-CD3 and anti-CD28 antibodies, and then incubated with B16-OVA tumor culture medium supernatant. 24 h later, the levels of phosphorylated Akt were determined by western blot. As shown in Fig. 1a, incubation with B16-OVA culture supernatant inhibited Akt phosphorylation, suggesting that Akt activation in T cells is inhibited by tumor secreted factors. To examine Akt activation state of T cells in tumor microenvironment *in vivo*, T cells were isolated from tumor draining lymph nodes (TDLN) and distant lymph nodes (LN) of B16-OVA tumor bearing mice, and Akt phosphorylation was detected. As we expected, the phosphorylation of Akt was down-regulated in TDLN comparing to distant LN (Fig. 1b).

### Over-expressing Akt in OT-1 cells enhances anti-tumor effect in mouse B16 melanoma model

**Fig. 2:**
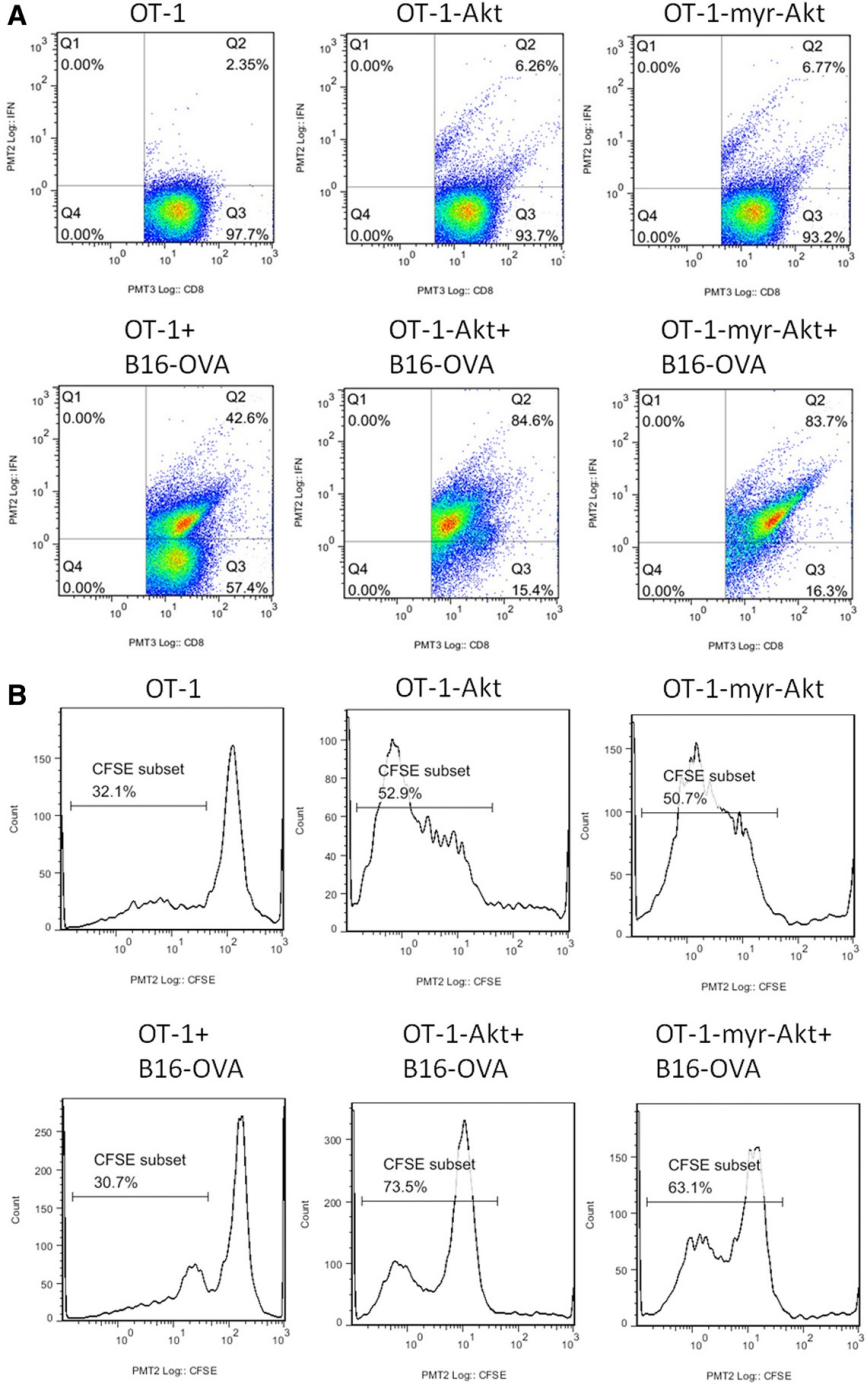
OT-1 cells transduced with Akt or myr-Akt display increased IFN-γ production and proliferation in the presence of B16-OVA tumor cells. **a** OT-1 cells transduced with control retroviruses or retroviruses encoding Akt or myr-Akt were incubated with or without B16-OVA tumor cells at an E:T ratio of 2:1, 24 h later, the cells were stained with anti-CD8 and anti-IFN-γ antibodies, and analyzed by flow cytometry. The analysis was carried out with gated CD8^+^ population. **b** OT-1 cells transduced with control retroviruses or retroviruses encoding Akt or myr-Akt were labeled with CFSE, and co-cultured with or without B16-OVA tumor cells at an E:T ratio of 2:1, 3 days later, the cells were stained with anti-CD8 antibody, and analyzed by flow cytometry. The analysis was carried out with CD8^+^ population

To examine if up-regulating Akt activation could increase the activity of T cells in the presence of tumor, we transduced OT-1 cells with retroviruses encoding wild type Akt (wtAkt) or a constitutively active form of Akt (myristoylated Akt, myr-Akt), and incubated the transduced OT-1 cells with B16-OVA tumor cells. As shown in Fig. 2a, when co-cultured with B16-OVA, OT-1 cells transduced with either wtAkt or myr-Akt produced more IFN-γ than OT-1 cells transduced with control retroviruses. In the proliferation assay, OT-1 cells transduced with control retroviruses slowed proliferation on exposure to B16-OVA tumor cells, whereas OT-1 cells transduced with either wtAkt or myr-Akt displayed higher proliferation rates when cultured alone, and further increased the percentage of proliferative populations when co-cultured with B16-OVA (Fig. 2b). Together, these data demonstrates that over-expressing Akt or myr-Akt could increase the cytokine production and proliferation of tumor specific T cells in the presence of tumor.

**Fig. 3:**
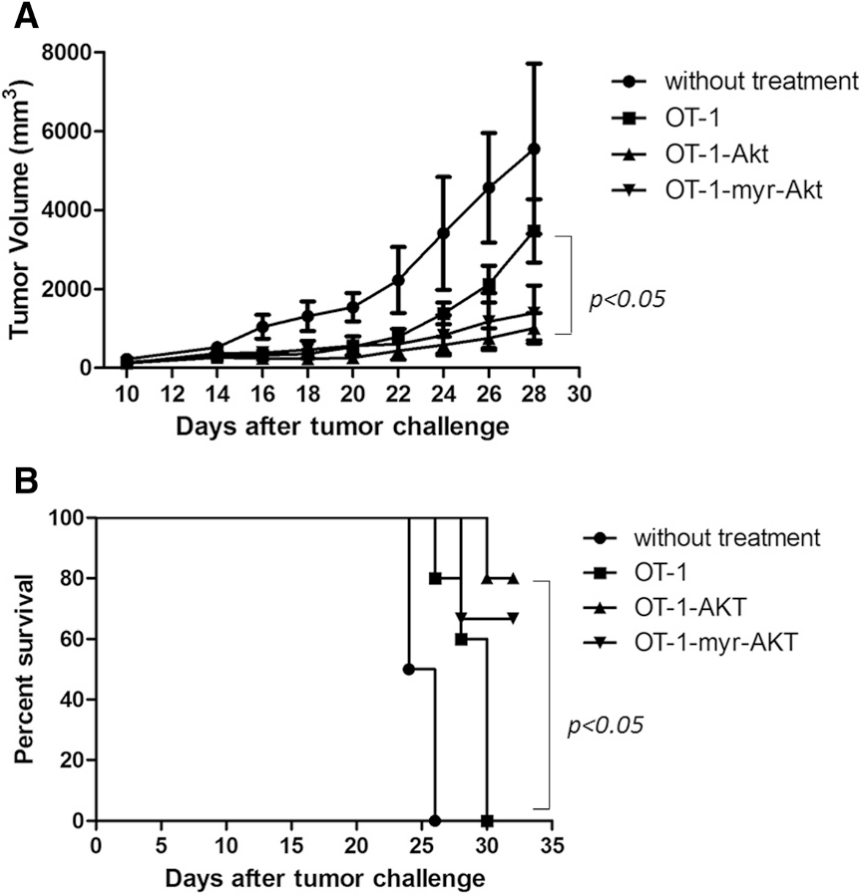
Adoptive transfer of OT-1 cells transduced with Akt can inhibit B16-OVA tumor growth and prolong mouse survival. OT-1 cells transduced with control retroviruses or retroviruses encoding Akt or myr-Akt were intratumorally injected into B16-OVA tumor bearing mice at 2x10^6^ cells/mouse at 11 days after tumor challenge, and the mice without treatment were used as control. Tumor growth was monitored every other day, and the death of mouse was defined when the tumor size reached 2 cm. **a** Line graph depicting the tumor volume of mice, statistical analysis was performed using two-way ANOVA. **b** Kaplan-Meier survival analysis of transduced OT-1 treated or untreated mice. Comparison of survival curves was made by Log-rank test

To investigate if transducing wtAkt or myr-Akt could improve the therapeutic effect of adoptively transferred T cells against tumor, C57BL/6 mice were inoculated with B16-OVA tumor cells subcutaneously, when tumor size reached ~6 mm, *in vitro* stimulated and expanded OT-1 cells transduced with control retroviruses or retroviruses encoding wtAkt or myr-Akt were intratumorally injected into mice. As shown in Fig. 3a, adoptive transfer of control OT-1 cells didn’t inhibit tumor growth comparing to the untreated group, whereas transducing wtAkt significantly increased the anti-tumor effect and prolonged mouse survival (Fig. 3b).

### Human T cells engineered to over-express Akt exert enhanced anti-tumor activity

**Fig. 4:**
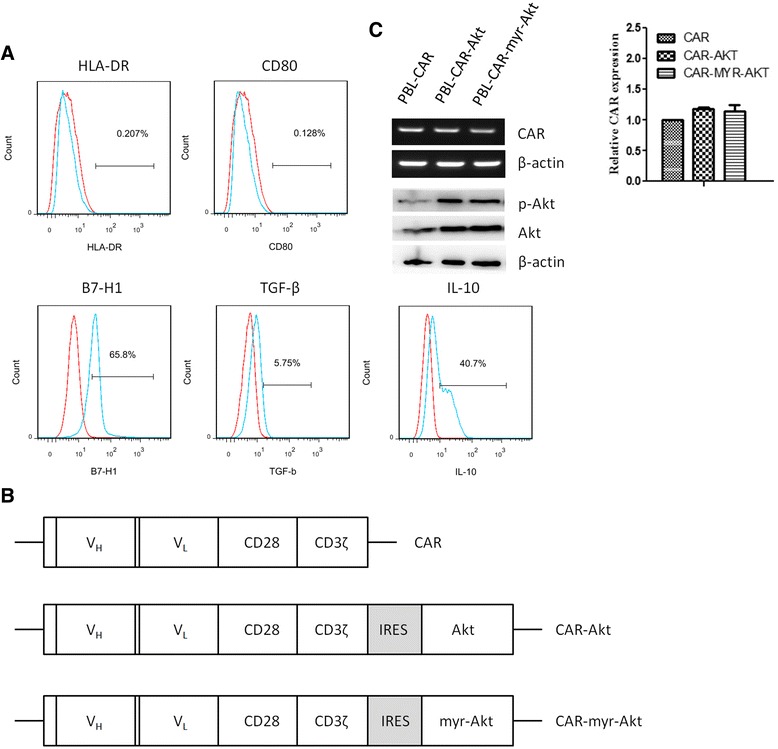
Engineer human peripheral blood lymphocytes (PBLs) to target prostate cancer cells. **a** Immunosuppressive phenotypes of human prostate cancer cell line PC3M were examined by staining with HLA-DR, CD80, B7-H1, TGF-β, and IL-10. **b** Different constructs used to transduce PBLs, V_H_, anti-human EpCAM immunoglobulin heavy chain variable region; V_L_, anti-human EpCAM immunoglobulin light chain variable region; CD28, part of the extracellular domain and the entire transmembrane and intracellular domains of CD28; CD3ζ, the cytoplasmic domain of CD3ζ; IRES, internal ribosome entry sequence. **c** Human PBLs were transduced with retroviruses encoding EpCAM specific CAR, CAR-Akt, or CAR-myr-Akt, at 5 days after transduction, the mRNA levels of transduced CAR were determined by RT-PCR and real-time PCR, and the protein levels of Akt, phosphorylated Akt were determined by western blot

To verify the above findings and further characterize the mechanisms by which wtAkt or myr-Akt transduced T cells resist tumor immunosuppression, we used the human prostate cancer model we established previously in our lab [16]. Prostate cancer cell line PC3M highly expresses EpCAM, and human peripheral blood lymphocytes (PBLs) were engineered to recognize PC3M by transducing with EpCAM specific chimeric antigen receptor (CAR) which consists of an anti-EpCAM scFv, part of the extracellular domain and the entire transmembrane and intracellular domains of CD28, and the cytoplasmic domain of CD3ζ. As shown in Fig. 4a, PC3M lacks expressions of HLA-DR and CD80, whereas expresses high levels of B7-H1, TGF-β, and IL-10. To enhance the anti-tumor activity of PBLs expressing EpCAM specific CAR against the immunosuppressive PC3M tumor, we generated CAR-Akt, or CAR-myr-Akt co-expressing retroviral vectors (Fig. 4b), and verified the transduction efficiency of CAR and Akt in PBLs by RT-PCR, real-time PCR and western blot, respectively (Fig. 4c). The transduction efficiency of CAR was also examined by staining with protein L, which is an immunoglobulin binding protein that can be used to determine CAR expression [17] (Additional file 1: Figure S1). Since it was reported that Akt inhibition promotes T cell differentiation to a memory phenotype [18], we evaluated CD62L expression in transduced T cells. As shown in Additional file 2: Figure S2, Akt over-expression didn’t down-regulate the level of CD62L.

**Fig. 5:**
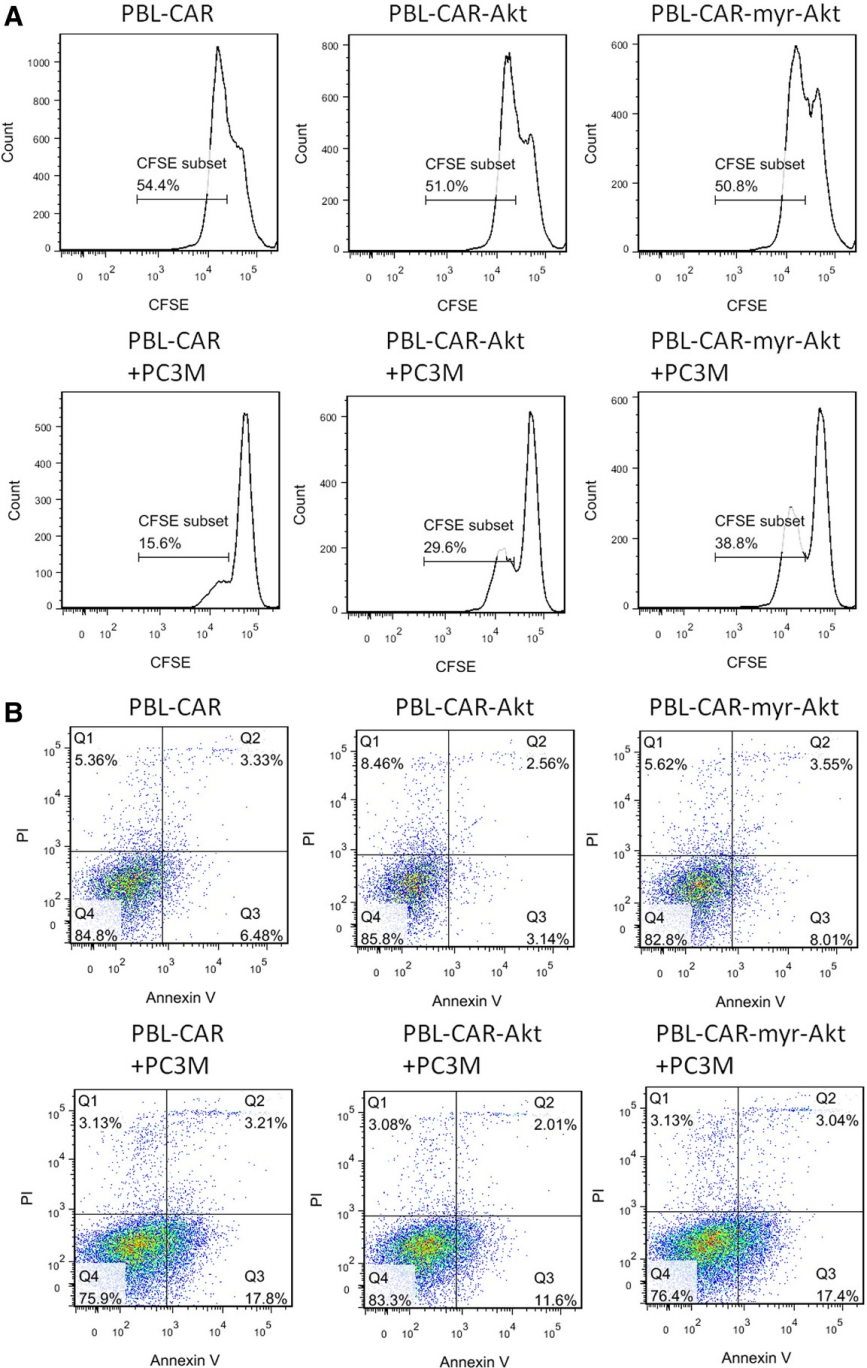
Over-expressing Akt increases the activity of CAR expressing PBLs in the presence of PC3M. PBLs transduced with CAR, CAR-Akt, or CAR-myr-Akt were co-cultured with or without PC3M at an E:T ratio of 2:1, 3 days later, cell proliferation was examined by CFSE assay (**a**), and cell apoptosis was determined by Annexin V/PI staining (**b**), the analysis was performed with gated CD8^+^ population

To determine if co-expressing Akt or myr-Akt could help PBLs resist tumor immunosuppression, PBLs transduced with CAR, CAR-Akt, or CAR-myr-Akt were co-cultured with PC3M tumor cells, 3 days later, T cell proliferation and apoptosis were examined. As shown in Fig. 5 and Additional file 3: Figure S3, T cell proliferations were all inhibited in the presence of tumor cells, however, the degree of inhibition was less in T cells co-expressing Akt or myr-Akt. In addition, incubation with tumor cells increased cell apoptosis in all groups, however, T cells co-expressing Akt displayed less apoptosis comparing to T cells expressing CAR only, and T cells transduced with myr-Akt displayed higher apoptosis rate than T cells transduced with wtAkt, suggesting that transducing wtAkt may be more beneficial than myr-Akt.

**Fig. 6:**
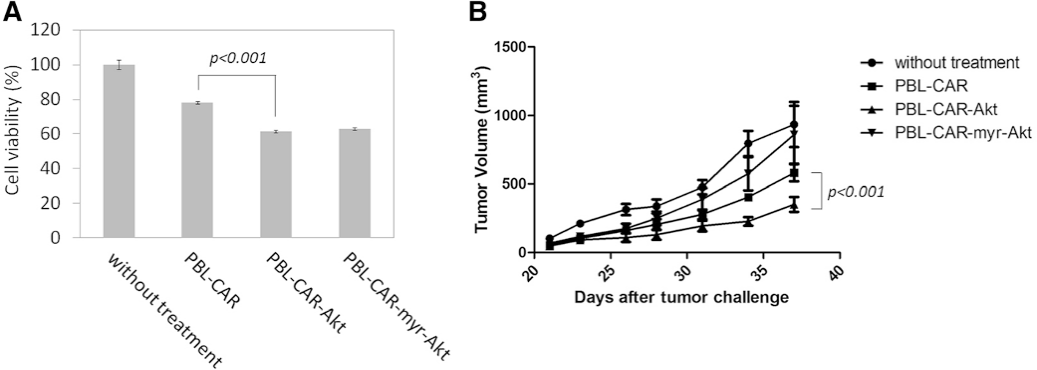
Over-expressing Akt enhances the anti-tumor efficacy of PBLs *in vitro* and *in vivo*. **a** PBLs transduced with CAR, CAR-Akt, or CAR-myr-Akt were incubated with PC3M-luc at an E:T ratio of 2:1, 24 h later, cell viability was determined by luminescence imaging, and the luminescence intensity was normalized to that of PC3M-luc cells without treatment. Statistical analysis was performed using student’s *t*-test. **b** NOD/SCID mice were subcutaneously injected with PC3M tumor cells at 6 × 10^6^ cells/mouse, at 21 days after tumor challenge, PBLs transduced with CAR, CAR-Akt, or CAR-myr-Akt were intratumorally injected into mice at 3 × 10^6^ cells/mouse, and the mice without treatment were used as control. Tumor volume was measured every few days, and statistical analysis was performed using two-way ANOVA

Next, we examined the anti-tumor effects of PBLs co-expressing Akt or myr-Akt both *in vitro* and *in vivo*. PBLs transduced with CAR, CAR-Akt, or CAR-myr-Akt were incubated with luciferase-expressing stable cell line PC3M-luc, 24 h later, cytotoxicity was determine by luminescence imaging. As shown in Fig. 6a, PBLs co-expressing Akt caused more cell lysis than PBLs expressing CAR only. In NOD/SCID mice, PC3M tumor cells were inoculated subcutaneously, when tumor size reached ~3 mm, PBLs transduced with CAR, CAR-Akt, or CAR-myr-Akt were injected intratumorally. As shown in Fig. 6b, PBLs co-expressing Akt significantly inhibited tumor growth comparing to PBLs expressing CAR only. These data indicates that Akt transduction can increase the activity of T cells in tumor environment and enhance the anti-tumor efficacy of adoptively transferred tumor specific T cells.

## Discussion

In this study, we show that Akt activity of T cells is down-regulated in tumor environment, and transducing Akt increases the proliferation, cytokine production, and anti-tumor effects of tumor specific T cells.

Akt phosphorylations in T cells incubated with tumor culture supernatant or T cells isolated from TDLN were inhibited (Fig. 1a and b), consistent with previous findings that the activation status of Akt in T cells is a primary determinant of T cell sensitivity to tumor immunosuppression [19]. It’s noticeable that OT-1 cells transduced with myr-Akt didn’t shown further enhanced effector functions than OT-1 cells transduced with wtAkt, which was also demonstrated in PC3M tumor model. It could be partly explained by the similar phosphorylation levels of Akt observed for T cells transduced with wtAkt and myr-Akt (Fig. 4c), also the increased apoptosis observed for PBLs transduced with myr-Akt cultured alone or in the presence of tumor cells (Fig. 5b). The increased apoptosis observed for PBLs transduced with myr-Akt is inconsistent with previous report that T cells expressing constitutively active Akt up-regulated anti-apoptotic molecules and displayed decreased apoptosis [20]. This difference could be caused by the different experimental system used in their study, in which the apoptosis was evaluated at 4 weeks after retrovirus transduction, whereas our study characterized T cells at 5 days after transduction.

The increased apoptosis observed for myr-Akt transduced T cells is contradictory to the well known pro-survival role of Akt, however, it was reported that Akt regulates Fas-mediated cell death through transcriptional activation of Fas receptor [21]. In addition, it has also been shown that Akt-dependent GSK inhibition regulates activation induced cell death (AICD) of T cells [22]. It’s possible that over-expressing constitutively active Akt increased AICD of T cells, thus dampened the therapeutic efficacy of tumor specific T cells. The fact that tumor specific T cells transduced with wtAkt displayed better effector functions than T cells transduced with myr-Akt actually makes this strategy more convenient for clinical translation. Although it has been reported that T cells expressing constitutively active Akt still require antigen stimulation to grow [11], transducing constitutively active Akt raises safety concerns for the application of this strategy. Our data supports over-expressing wtAkt to enhance the therapeutic effects of adoptively transferred tumor specific T cells, which serves as a foundation for broader clinical translation.

OT-1 cells transduced with Akt produced IFN-γ and increased the percentage of proliferative cells on exposure to B16-OVA, indicating the specific activation of T cells by tumor. In contrast, PBLs expressing CAR-Akt co-cultured with PC3M displayed decreased proliferation and increased apoptosis comparing to cultured alone, suggesting that PC3M model is more immunosuppressive, and up-regulating Akt is not sufficient to enable T cells to completely resist the suppression. Despite this, PBLs expressing CAR-Akt increased anti-tumor effects both *in vitro* and *in vivo*, comparing to PBLs expressing CAR only. It’s worth to mention that the EpCAM specific CAR we used consists of both CD28 and CD3ζ signaling domains, thus the increased cytotoxicity displayed by PBLs co-expressing Akt is probably due to resistance to inhibitory signaling conferred by Akt activation.

The recent report by Crompton et al. found that inhibition of Akt enabled TIL a memory T cell signature with enhanced anti-tumor effect [23]. TILs isolated for adoptive transfer normally undergo extensive expansion process *in vitro*, and display a terminally-differentiated phenotype with short telomere and diminished anti-tumor activity. In their study, Akt inhibitor was added only in the expansion process, and they found that TILs expanded with Akt inhibition persisted longer and exerted better anti-tumor effects. Comparing to their study, we used OT-1 T cells or PBLs stimulated and expanded *in vitro*, which displayed a CD62L^+^ phenotype after the short expansion process (Additional file 2: Figure S2). Akt is necessary to induce and sustain effector functions of T cell [18, 24], comparing to their study trying to enhance the persistence of T cells by inhibition of Akt, we tried to enhance the effector functions of T cells by over-expressing Akt, these two studies take different angles to manipulate T cells to enhance the anti-tumor efficacy, and are not contradictory to each other, and it’s worth further study to determine which means is superior to the other. In addition, in their work, tumor bearing mice were pretreated with total body irradiation, adjuvant vaccine, and IL-2 in conjunction with cell therapy, these preparations could ameliorate tumor immunosuppression and enhance the anti-tumor effects of cell therapy, however, severe adverse effects have been reported. In contrast, we used adoptive cell therapy alone to treat tumor, and over-expressing Akt could enable T cells to resist tumor immunosuppression, which is the major advantage of our strategy.

## Conclusions

In summary, our data demonstrates that over-expressing Akt in tumor specific T cells could improve the proliferation, cytokine production, and cytotoxicity of T cells in the presence of tumor. Comparing to strategies designed to counteract various immunosuppressive mechanisms employed by tumor, transducing Akt in T cells provides a new approach for tumor immunotherapy, and has important implications for improving the therapeutic effects of adoptive T cell transfer.

## Additional files

Additional file 1: Figure S1. Transduction efficiency of EpCAM CAR. PBLs were transduced with CAR, CAR-Akt, or CAR-myr-Akt, 4 days later, CAR expression was detected by biotinylated Protein L and PE conjugated streptavidin. A) FACS histogram B) Quantification of transduction efficiency of PBLs from 3 healthy donors. The untransduced PBLs were used as the negative control. (JPEG 82 kb)
Additional file 2: Figure S2. CD62L expression on transduced PBLs. PBLs were transduced with CAR, CAR-Akt, or CAR-myr-Akt, 4 days later, the cells were stained with CD4, CD8, and CD62L. A) FACS histogram B) Quantification of CD62L expression on CD4^+^ or CD8^+^ cells from 3 healthy donors. (JPEG 790 kb)
Additional file 3: Figure S3. Quantification of cell proliferation and apoptosis of transduced PBLs. PBLs transduced with CAR, CAR-Akt, or CAR-myr-Akt were co-cultured with or without PC3M at an E:T ratio of 2:1, 3 days later, cell proliferation and apoptosis were determined. A) Percent of CFSE^+^ cells in CD8^+^ population B) Percent of Annexin V^+^ cells in CD8^+^ population. Experiments were repeated for 3 times, and statistical analysis was performed using student’s *t*-test. (JPEG 99 kb)
