# Supplementary figures and images for: Over-expressing Akt in T cells to resist tumor immunosuppression and increase anti-tumor activity
Source: BMC Cancer. 2015 Aug 27;15:603. doi: 10.1186/s12885-015-1611-4 (PMC4550078; doi:10.1186/s12885-015-1611-4)

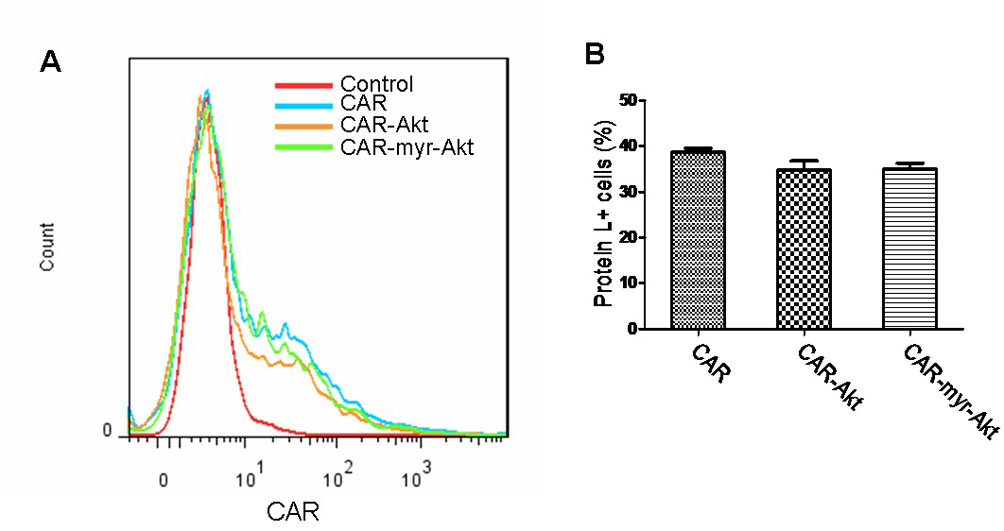

Supplement: Additional file 1: Figure S1. — Transduction efficiency of EpCAM CAR. PBLs were transduced with CAR, CAR-Akt, or CAR-myr-Akt, 4 days later, CAR expression was detected by biotinylated Protein L and PE conjugated streptavidin. A) FACS histogram B) Quantification of transduction efficiency of PBLs from 3 healthy donors. The untransduced PBLs were used as the negative control. (JPEG 82 kb) [file 12885_2015_1611_MOESM1_ESM.jpeg]

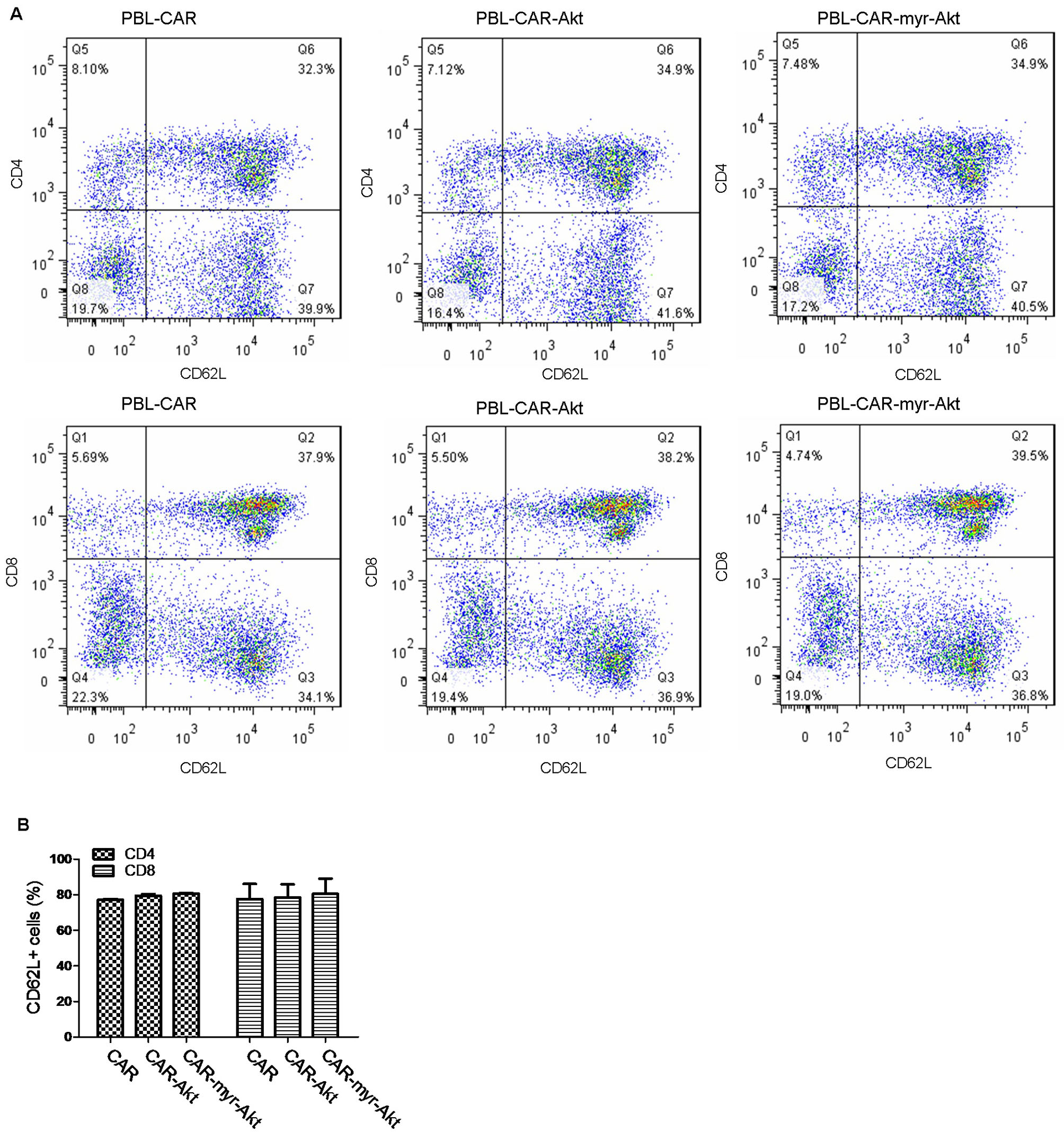

Supplement: Additional file 2: Figure S2. — CD62L expression on transduced PBLs. PBLs were transduced with CAR, CAR-Akt, or CAR-myr-Akt, 4 days later, the cells were stained with CD4, CD8, and CD62L. A) FACS histogram B) Quantification of CD62L expression on CD4+ or CD8+ cells from 3 healthy donors. (JPEG 790 kb) [file 12885_2015_1611_MOESM2_ESM.jpeg]

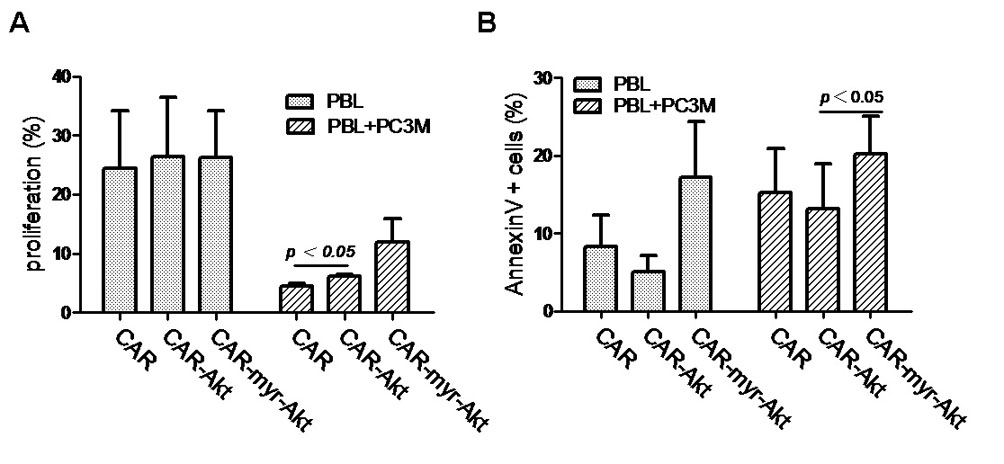

Supplement: Additional file 3: Figure S3. — Quantification of cell proliferation and apoptosis of transduced PBLs. PBLs transduced with CAR, CAR-Akt, or CAR-myr-Akt were co-cultured with or without PC3M at an E:T ratio of 2:1, 3 days later, cell proliferation and apoptosis were determined. A) Percent of CFSE+ cells in CD8+ population B) Percent of Annexin V+ cells in CD8+ population. Experiments were repeated for 3 times, and statistical analysis was performed using student’s t-test. (JPEG 99 kb) [file 12885_2015_1611_MOESM3_ESM.jpeg]
